# Perceptions and Realities for Distal Freehand Interlocking of Intramedullary Nails

**DOI:** 10.1155/2015/834582

**Published:** 2015-04-05

**Authors:** Robert F. Ostrum

**Affiliations:** Department of Orthopaedic Surgery, University of North Carolina, 3160 Bioinformatics Building, CB 7055, Chapel Hill, NC 27599, USA

## Abstract

There is a perception that distal freehand interlocking (DFHI) of intramedullary nails can be difficult and time consuming. This study consists of a survey of surgeons' practices for DFHI screws and their reasons for not using this technique. A survey was sent to 1400 orthopaedic surgeons who were asked to agree or disagree with statements regarding the difficulty and indications for the usage of distal freehand interlocking screws. The results were analyzed by practice demographics, resident availability, and completion of an orthopaedic trauma fellowship. Overall, 316 surgeons (22.6%) responded to the survey. Fellowship trained surgeons were 60% less likely to find DFHI difficult when compared to nonfellowship surgeons and surgeons with residents were 76% less likely to perceive DFHI as difficult than surgeons without residents. In all groups, 40–43% of surgeons used distal interlocking based on their comfort with the technique and not the fracture pattern. Distal freehand interlocking is perceived as difficult by community orthopaedic surgeons without residents and surgeons who have not done an orthopaedic trauma fellowship. Forty percent of surgeons based their usage of DFHI screws on their comfort with the technique and not the fracture pattern.

## 1. Introduction

Proximal femur fractures are a common injury treated by orthopaedic surgeons. Currently, a large number are surgically treated with an intramedullary nail (IMN). Which type of IMN is employed appears to be based on surgeon preference and other associated variables. Starr et al. compared piriformis entry IMNs to trochanteric IMNs in the treatment of proximal femur fractures. The authors found no differences in any of the surgical parameters that they looked at nor did they find that either nail was preferable for functional outcome [1]. Other authors have looked at the use of trochanteric entry, cephalomedullary devices such as the Gamma nail (Stryker, Mahwah, New Jersey) in the treatment of subtrochanteric fractures [2–14]. In two reviews of the Cochrane Database of Systematic Reviews, Parker and Handoll found that when comparing a short IMN to a sliding hip screw (SHS) in the treatment of extracapsular femur fractures the short IMNs had a higher complication rate [2, 3]. Hesse and Gächter demonstrated a 16% general complication rate and an 8% implant related complication rate when they studied the use of a short Gamma nail for trochanteric fractures [4, 5]. Thigh pain and refracture at the tip of the short IMN have been the most common complications seen with use of a short intramedullary implant [2–14]. Some orthopaedic surgeons have progressed to using a long intramedullary device to treat proximal femur fractures to avoid the complications seen with short nails and because they believe that these fractures, often associated with osteoporosis, represent a pathologic fracture [15]. The use of a short IMN allows the surgeon to use an outrigger device attached to the insertion handle to insert the interlocking screws in the distal end of the IMN. However, the use of a long intramedullary nail requires the surgeon to employ the technique of distal freehand interlocking screw placement. This cannot be done with the assistance of a jig and requires some level of surgical expertise and additional operating room and fluoroscopic time.

The purpose of this study was to look at the practice patterns and preferences of orthopaedic surgeons when treating a proximal femur fracture with an intramedullary nail and to determine whether the use of distal freehand interlocking of a long nail was driven by the fracture pattern or surgeon comfort with the technique.

## 2. Materials and Methods

**Figure 1:**
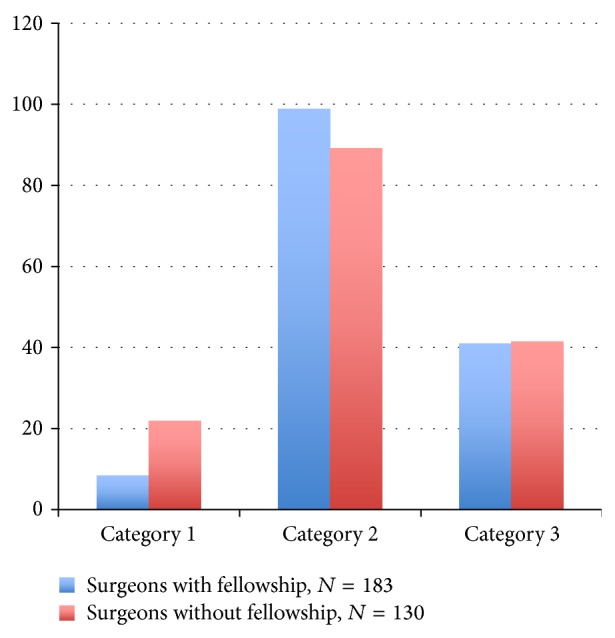
Percent agreement comparison of fellowship and nonfellowship trained orthopaedic surgeons.

**Figure 2:**
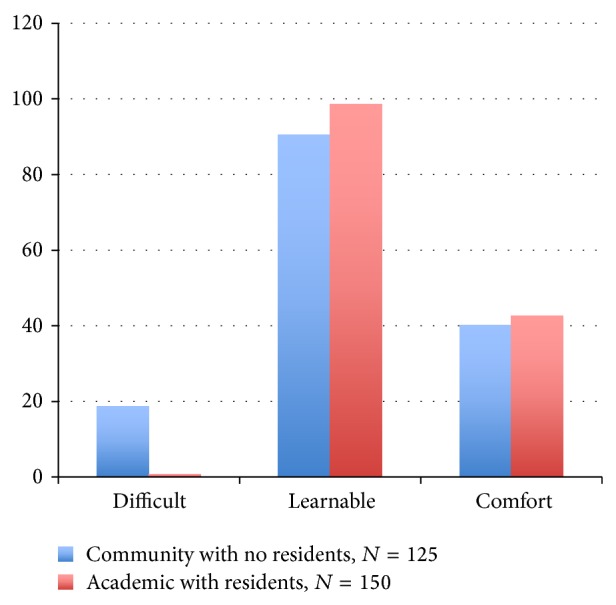
Percent agreement comparison of community surgeons without residents versus academic surgeons with residents.

**Figure 3:**
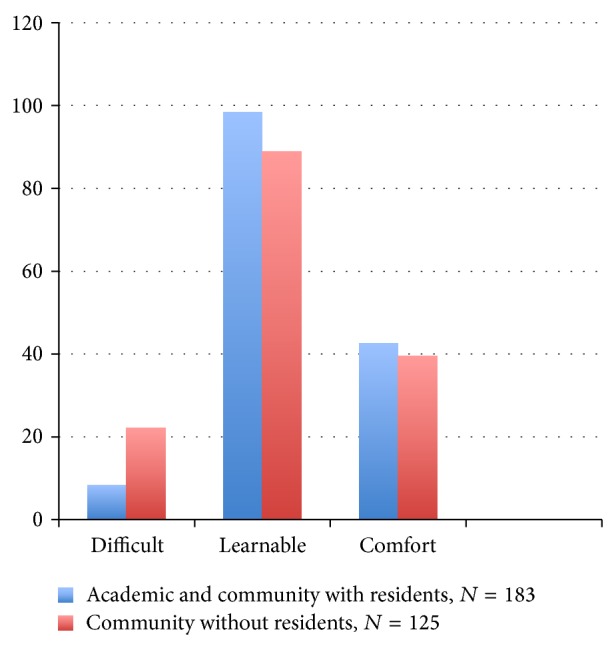
Percent agreement comparison of all surgeons with residents versus community surgeons without residents.

An anonymous, voluntary survey was sent out to 1400 orthopaedic surgeons through an Internet link via SurveyMonkey (https://www.surveymonkey.com/) to the Pennsylvania Orthopaedic Society, New Jersey Orthopaedic Society, and the Orthopaedic Trauma Association. There were a total of 316 (22.6%) responses received out of the approximately 1400 surveys that were sent. The survey included the following:* (1) the utilization of distal, freehand interlocking screws can be difficult and is often time consuming; (2) the use of distal, freehand interlocking screws is an easy, learnable technique; (3) my choice to use distal, freehand interlocking screws is based on my comfort with the technique and not the fracture pattern*. Demographic data was collected on the respondents that included their extent of orthopaedic training, that is, those who had done an orthopaedic trauma fellowship (WF) and those who did not have a fellowship (NF) (Figure 1). Further, the type of practice was recorded and categorized into the following: those surgeons practicing in the community with residents (CWR), those community surgeons without residents (CNR), and those in academic practice with residents (AWR) (Figures 2 and 3). Responses were analyzed by Pearson *X* ^2^ or Fisher exact test in accordance with distributional assumptions and filtered by the type of practice and presence or absence of trauma fellowship. Separate multiple logistic regression models were fit to utilization of distal freehand interlocking screws, ease of the DFHI technique, and choice to use distal freehand interlocking screws.

## 3. Results

Of the 316 respondents, the practice groups included 125 surgeons who were community surgeons without residents (CNR), 33 were community surgeons with residents (CWR), 150 were in academic practice with residents (AWR), and 8 surgeons listed their practice as other and were not included in the analysis. When analyzing the sample by fellowship training there were 183 surgeons who had finished an orthopaedic trauma fellowship (WF) and 130 who had not done a fellowship (NF); 3 did not answer and were not included in the analysis. The analyses were done comparing the CWR, the CNR, and the AWR groups to each other and then the results for the WF and NF groups were compared. With Bonferroni correction to *P* < 0.003, the CWR and AWR groups had no significant differences on any of the variables considered, nor did the CWR and CNR physicians. When analyzing whether distal freehand interlocking was a difficult and time consuming procedure, there were no differences when comparing the AWR and the CWR groups. However, the CNR group was significantly more likely than the AWR group (*P* = 0.001) to perceive distal interlocking as difficult and not easily learned as was the NF group compared to the WF group (*P* = 0.001). The greatest disparity was seen when comparing the AWR group to the CNR group and the NF group to the WF group who were dissimilar in their perceptions of the difficulty of distal interlocking and whether this was an easily learned technique (*P* = 0.002). Multiple logistic regression results show fellowship trained surgeons to be 60% (95% CI, 18–81%) less likely (*P* = 0.01) to find DFHI difficult after adjusting for community versus academic practice and whether or not residents were present. Multiple logistic regression results further showed that surgeons with residents were 76% (95% CI = 0.05–1.09) less likely (*P* = 0.07) to perceive DFHI as difficult when compared to surgeons without residents. When questioned about whether “DFHI is an easy, learnable technique,” the WF surgeons were 6.8 times more likely (95% CI = 1.47–31.17) to agree that DFHI is easily learned than the NF surgeons. As for “my choice to use DFHI screws is based on my comfort with the technique and not the fracture pattern,” 41% of all surgeons used distal freehand interlocking based on their comfort with the technique and not the fracture pattern.

## 4. Conclusions

This study demonstrated that surgeons who work with residents and those that had done an orthopaedic trauma fellowship found distal freehand interlocking an easily learned technique and that it was employed by them more often than those surgeons without residents or fellowship training. The pendulum seems to have swung in the intramedullary nailing direction for the treatment of pertrochanteric fractures. Anglen and Weinstein showed that, among those orthopaedists taking Part II of the American Board of Orthopaedic Surgery certification examination in 2006, 67% preferred an intramedullary nail over a sliding hip screw for fixation of a trochanteric fracture versus only 3% intramedullary fixation in 1999 [16]. The reasons for this paradigm shift are unclear as the literature supports no clear advantage to the short IM nail over a SHS and to the contrary short intramedullary nailing has a higher complication rate than plate fixation [2–14]. Mortality and functional outcome were the same independent of the implant utilized [2]. Hesse and Gächter reported an 8% of implant related complications when treating proximal femur fractures with a short Gamma nail [4, 5], while Madsen et al. reported a reoperation rate of 8% and a 4% implant related fractured femur rate was reported with the use of short Gamma nail in the treatment of unstable pertrochanteric fractures [6]. All of these studies as well as other comparative studies demonstrated little benefit to the short IM nail versus the SHS, but all showed a higher complication rate with the short intramedullary nail [2–14]. Although there are many papers that examine the differences between a short IMHS and a SHS, there are none comparing a long intramedullary nail to a short intramedullary nail in the treatment of proximal femur fractures.

Short nails are commonly employed due to the fact that the distal screws can be placed using the insertion jig. Kempf et al. tried to make a distal targeting device that attached to the C-arm or the nail and allowed for simple insertion of distal interlocking screws in long nails [17]. These apparatuses were neither simple to use nor reliable and in the early 1990s freehand distal interlocking was introduced and became the preferred method for the insertion of distal interlocking screws.

The limitations of this study are inherent in the fact that there was only a 22.6% response rate to the survey; however, a good cross section of the orthopaedic community was represented in appropriate proportions. The availability of short IM nails, which do not require freehand interlocking, has made these implants an easy solution for treating femoral shaft fractures by those surgeons uncomfortable with the freehand technique. In this study the perceived difficulty of distal freehand interlocking led to 40% of surgeons making decisions on appropriate fracture implant based on their ability (or inability) to place these screws distally through the nail. This is not uncommon in orthopaedics as surgeons often shy away from techniques that they are not comfortable with or those with which they have little experience. The perception that distal freehand interlocking was difficult and time consuming was seen predominantly in the nonfellowship trained group (NF) and the community group without residents (CNR) when compared to those with fellowship (WF) and those with residents (CWR, AWR), respectively. The author would like to recommend that surgeons who feel that they are uncomfortable with distal freehand interlocking partake of any number of courses that include hands on workshops to learn this technique and be able to employ it as necessary.

Distal freehand interlocking of long femoral nails has now become the accepted method of screw insertion. Surgeons with fellowship trauma training and those with residents agreed that distal freehand interlocking was not a difficult technique and was easily learned when compared to those surgeons who had neither fellowship training nor residents. There is further support that this freehand technique is simple and easily learned as evidenced by the respective times for distal locking from studies from the late 1980s and early 1990s to the present [18–20].

In a 1987 paper Levin et al. looked at radiation exposure with intramedullary nail insertion and found that distal freehand interlocking used an average of 2.7 minutes of fluoroscopy (0.6–6.6) [18]. In 1993 Sanders et al. demonstrated that it took an average of 6.26 minutes of fluoroscopy time and 151 minutes of OR time to perform femoral intramedullary nailing [19]. Further, they found that the fluoroscopic time required for femoral nailing was 2.6 times greater when distal freehand interlocking was added, compared to those without distal locking [19]. Chan et al. recently showed that freehand distal interlocking screw set-up time was 105 seconds with an associated 10 seconds of fluoroscopy time [20]. To insert a single interlocking screw required 342 seconds of OR time and 18 seconds of fluoroscopy time [20].

Clearly we have advanced in the employment of distal freehand interlocking to a point where time and radiation exposure have been cut to fractions of the time it used to take to perform this procedure 15–20 years ago. This further supports the fact that with experience and development of surgical technique this is an easily learned technique that could be applied by most surgeons when treating these fractures. The decision to place a long or short intramedullary nail should be based on the fracture's location and morphology as well as the overall quality of the patient's bone. Distal freehand interlocking screws should be used when there is obvious or implied axial or rotational instability. Brumback et al. demonstrated that it is difficult intraoperatively to determine stability as over 10% of patients treated without distal screws in their study had loss of fixation and reduction [21].

In conclusion, the perception that distal freehand interlocking is difficult and that usage of this technique with long nails is based on comfort with the technique and not the fracture pattern is reality and is seen especially in the community orthopaedists without residents and those surgeons without orthopaedic trauma fellowship training. Distal freehand interlocking appears to be an easily learned technique with operating room and fluoroscopy times showing a huge decrease when compared to data of 20 years ago. The use of a long or short intramedullary nail for proximal femur fractures should probably not be based on fear or the perceived difficulties associated with distal freehand screw insertion but rather on the fracture pattern and the patient's bone quality.
